# Summary of best evidence on parental involvement in neonatal intensive care units: integrating family-centered and family-integrated care models

**DOI:** 10.1080/16549716.2026.2653286

**Published:** 2026-05-05

**Authors:** Jiahui He, Ningqing Chen, Youyu He, Qiumei Wang

**Affiliations:** a Department of Neonatology, Obstetrics & Gynecology Hospital of Fudan University, Shanghai Key Lab of Reproduction and Development, Shanghai Key Lab of Female Reproductive Endocrine Related Diseases, Shanghai, China; b Department of Nursing, Obstetrics & Gynecology Hospital of Fudan University, Shanghai Key Lab of Reproduction and Development, Shanghai Key Lab of Female Reproductive Endocrine Related Diseases, Shanghai, China

**Keywords:** Parental involvement, family-centered care, family-integrated care, neonatal intensive care units, best evidence

## Abstract

Family-Centered Care (FCC) and Family-Integrated Care (FICare) are widely adopted models in Neonatal Intensive Care Units (NICUs), designed to foster parental involvement and support both neonatal and family outcomes. This review synthesizes and critically appraises the best available evidence on FCC and FICare interventions to inform their implementation, adaptation, and scale-up across diverse health systems and cultural contexts. Guided by the 6S evidence model, a top-down search identified relevant guidelines, best practices, evidence summaries, expert consensus statements, systematic reviews, and meta-analyses published up to 20 May 2025. Two reviewers independently performed study selection, methodological appraisal, and data extraction, with evidence graded using the Joanna Briggs Institute system. A total of 25 publications were included: three clinical guidelines, three best practice documents, four expert consensus statements, and fifteen systematic reviews and meta-analyses. Synthesis revealed seven key domains: core components of FCC/FICare models, implementation strategies, clinical outcomes, safety considerations, cultural adaptability, ethical considerations, and digital health applications. From these, 28 high-quality recommendations were formulated. Overall, FCC and FICare consistently improved neonatal outcomes and enhanced family well-being. Structured parent education, psychosocial support, environmental optimization, and interdisciplinary collaboration emerged as essential elements for effective implementation. Digital health tools may serve as valuable adjuncts but should complement rather than replace relational and presence-based care. Addressing cultural, ethical, and organizational barriers is critical to achieving equitable and sustainable integration. These findings reinforce FCC/FICare as a foundational model for advancing neonatal care globally.

## Paper context

- **Main findings:** This review synthesizes high-level evidence on FCC and FICare in NICUs, highlighting seven key domains for effective implementation. Structured parental involvement consistently improves neonatal outcomes and parental competence and reduces psychological stress.
- **Added knowledge:** Integrating clinical guidelines, best practice documents, expert consensus statements, and systematic reviews/meta-analyses using the 6S model and the JBI grading framework, the review generates 28 actionable recommendations, offering a consolidated framework for effective, safe, and culturally adaptable FCC/FICare implementation.
- **Global health impact for policy and action:** The findings offer actionable guidance for policymakers and health system leaders to support the adaptation and scaling up of FCC/FICare across diverse health system and cultural contexts. These recommendations can inform workforce training, service design, and resource allocation, contributing to more equitable and family-centered neonatal care globally.

## Background

Globally, NICUs are facing mounting challenges, as the number of preterm births continues to rise. In 2020, an estimated 13.4 million infants were born before the 37 weeks of gestation. At the same time, there is an increasing burden of complex health issues among surviving newborns, including respiratory complications and neurodevelopmental impairments [1,2]. While medical advancements have significantly improved survival rates for critically ill newborns, emerging evidence underscores that optimal neurodevelopmental outcomes depend not only on clinical interventions but also on active family engagement [3]. FCC is a collaborative healthcare model emphasizing dignity, respect, and active family involvement in care planning and delivery. Core principles include information sharing, emotional and physical support, and family participation in decision-making. It recognizes all family members as care recipients, adapting to diverse family structures [4,5]. FICare is an advanced FCC model in neonatal settings, empowering parents to co-deliver clinical care, improving healthcare efficiency, staff satisfaction, and care quality. Both frameworks prioritize mutual respect, shared responsibility, and psychosocial-cultural sensitivity [4,5]. The FCC and FICare models have been widely advocated to address the need for enhanced parental engagement in neonatal intensive care, positioning parents as active collaborators in care delivery rather than passive observers [6]. Despite broad recognition of their theoretical benefits, substantial challenges persist in how these models are operationalized across healthcare systems, leading to fragmented implementation and unequal access to family-driven care [7,8].

Existing research on FICare and FCC in NICUs predominantly focuses on isolated aspects, such as parental stress reduction or short-term neonatal outcomes, while largely overlooking the need for holistic frameworks that integrate implementation strategies, long-term clinical effectiveness, ethical considerations, and contextual adaptability [9,10]. The urgency to bridge this gap is amplified by evolving healthcare paradigms and socioeconomic realities. Modern neonatal care increasingly prioritizes patient- and family-reported outcomes, with growing legal and ethical imperatives to involve families in decision-making [11]. Concurrently, economic analyses indicate that prolonged NICU stays contribute disproportionately to healthcare costs [12]. Moreover, parent–infant interactions within the NICU are complex and are shaped by cultural, emotional, medical, and developmental factors. Accordingly, outcome measures must be adaptable to individual variability, rapid infant developmental changes, and the sociocultural context of care [13]. These intersecting challenges demand a cohesive synthesis of high-quality evidence to guide actionable, context-sensitive solutions.

To address these challenges, this review synthesizes evidence from guidelines, best practices, expert consensus statements, systematic reviews, and meta-analyses to address persistent gaps in implementing the FCC and FICare models within NICUs, utilizing the 6S evidence hierarchy and the JBI framework. This study aims to support clinical practice by systematically identifying the best available evidence on integrating FCC and FICare models.

## Materials and methods

### Study design

**Figure 1.**
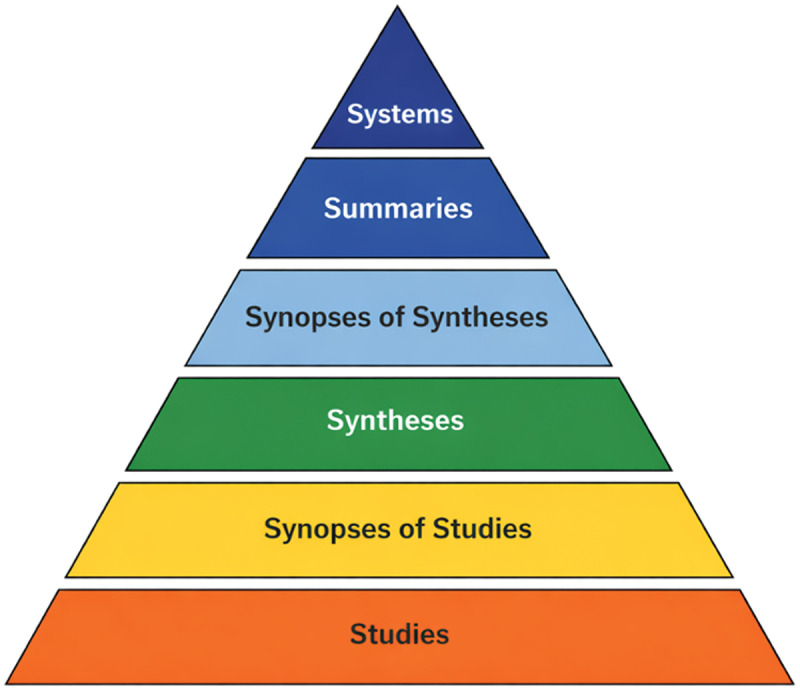
The 6S hierarchy of evidence adapted from DiCenso, Baley L, Haynes RB. Evid based Nurs. 2009;12(4):99–101.

This study was conducted as a structured best-evidence summary aimed at synthesizing high-level, pre-appraised evidence to inform clinical practice in neonatal intensive care units (NICUs). The methodology was guided by the 6S hierarchy of evidence [14], which advocates a top-down approach to identifying the highest levels of synthesized evidence to support evidence-based decision-making (Figure 1). This review focused on pre-processed evidence sources, including clinical practice guidelines, best practice documents, expert consensus statements, and systematic reviews or meta-analyses. Evidence identification, appraisal, grading, and synthesis, as well as recommendation development, were conducted in accordance with the Joanna Briggs Institute (JBI) framework for evidence-based healthcare (see Supplementary Table S1 for the full workflow).

### Search strategy

Based on the ‘6S’ hierarchy of evidence, the process of evidence retrieval follows a top-down approach, starting from the highest level of synthesized resources. Evidence was retrieved from a range of reputable clinical guideline repositories and evidence-based platforms, including international and national organizations, such as BMJ Best Practice, UpToDate, the National Institute for Health and Care Excellence (NICE), the Agency for Healthcare Research and Quality (AHRQ), the Guideline International Network (GIN), the Canadian Medical Association (CMA Infobase), the Scottish Intercollegiate Guidelines Network (SIGN), the China Guideline Clearinghouse (CGC), the World Health Organization (WHO), the New Zealand Guidelines Group (NZGG), and the Registered Nurses’ Association of Ontario (RNAO). Additional searches were conducted in evidence-based medical databases, such as the Trip Database, the JBI Evidence-Based Healthcare Database (Australia), and the Cochrane Library, as well as major literature databases, including PubMed, EBSCO-CINAHL, Web of Science, CNKI, Wanfang Data, VIP, and Sinomed.

A comprehensive search strategy was constructed by combining Medical Subject Headings (MeSH) with relevant free-text terms, focusing on three core elements: (1) NICU or neonates/newborns, (2) family engagement approaches, such as parental involvement, FCC, or FICare, and (3) restrictions based on literature type. The detailed search terms included: (neonate* OR infant* OR newborn* NICU or Neonatal Intensive Care Unit) AND (parental involvement OR family ward OR family-centered care OR FCC OR family-integrated care OR FIC) AND (guideline* OR ‘practice guideline’ OR routine* OR ‘recommended practice’ OR ‘standard’ OR ‘recommendation’ OR ‘evidence summary’ OR consensus* OR ‘systematic review’ OR ‘meta-analysis’). No geographic restrictions were applied during the search process to ensure global representation of evidence. The search time frame is from database inception to 20 May 2025.

### Inclusion and exclusion criteria of evidences

Inclusion and exclusion criteria were predefined to ensure relevance to the review objective and consistency with the 6S hierarchy of evidence. Evidence was eligible if it: (i) involved neonates admitted to NICUs and their families; (ii) addressed parental participation, Family-Centered Care (FCC), or Family-Integrated Care (FICare); (iii) represented high-level evidence, including clinical guidelines, best practice documents, evidence summaries, systematic reviews, meta-analyses, or expert consensus statements; and (iv) was published in English or Chinese. For mixed-methods systematic reviews, only quantitative components were extracted and appraised. Evidence was excluded if it consisted of conference abstracts, guideline commentaries, research protocols, or officially superseded guidelines, or if full texts were inaccessible or data were incomplete. Best practice documents and guidelines were included only when supported by explicit evidence sources to maintain methodological rigor.

### Study selection

Two reviewers with training in evidence-based medicine independently screened all retrieved records using predefined inclusion and exclusion criteria. Screening was conducted in several stages, including duplicate removal, title and abstract screening, and full-text review for eligibility. Disagreements at any stage were resolved through discussion, and a third reviewer was consulted if consensus could not be reached. The study selection process was conducted in accordance with the PRISMA 2020 guidelines. The PRISMA flow diagram and checklist are provided as separate files alongside the manuscript.

### Literature quality evaluation

The quality of the included evidence was appraised using validated tools matched to the type of literature. For clinical practice guidelines, the AGREE II instrument was employed, which evaluated 23 items across six domains, such as scope and purpose, stakeholder involvement, and editorial independence. Each item is rated on a 7-point Likert scale, and domain scores are calculated as standardized percentages to ensure comparability [15,16]. Systematic reviews and meta-analyses were assessed using the 2016 version of the JBI Critical Appraisal Checklist for Systematic Reviews and Research Syntheses, which includes 11 items designed to evaluate methodological rigor, clarity of review objectives, and appropriateness of synthesis methods [17]. For expert consensus documents, the 2016 Expert Consensus Standards from the Joanna Briggs Institute were applied, consisting of six appraisal criteria focused on the clarity of purpose, source of evidence, and consensus process [18]. In terms of best practice evidence, the original underpinning research was reviewed, and the corresponding quality appraisal tools were selected according to the specific study design, ensuring the credibility and reliability of the summarized evidence. Two reviewers independently conducted the quality assessments, with a third reviewer resolving any disagreements. Conflicting findings were addressed by prioritizing higher-quality, evidence-based, and recent publications.

### Data extraction

After quality assessment, two reviewers independently extracted data using a standardized form, with each reviewer blinded to the other’s entries. Extracted information included study characteristics (author, affiliation, publication year, evidence type, source, and study focus) and key findings or recommendations. Extracted evidence was organized thematically into conceptual domains to support structured synthesis and transparent reporting.

### Principles for rating the quality of evidence and strength of recommendations

The evidence included was assessed and rated using the JBI grading system for evidence and recommendations [19]. Evidence grades are categorized into five levels (1–5) based on the study design. Additionally, following the JBI’s FAME framework, the evidence is evaluated for feasibility, appropriateness, meaningfulness, and effectiveness. Based on this evaluation, the evidence is assigned a recommendation level: A-level indicating a strong recommendation, and B-level indicating a weak recommendation.

## Results

### Overview of the included studies

**Figure 2.**
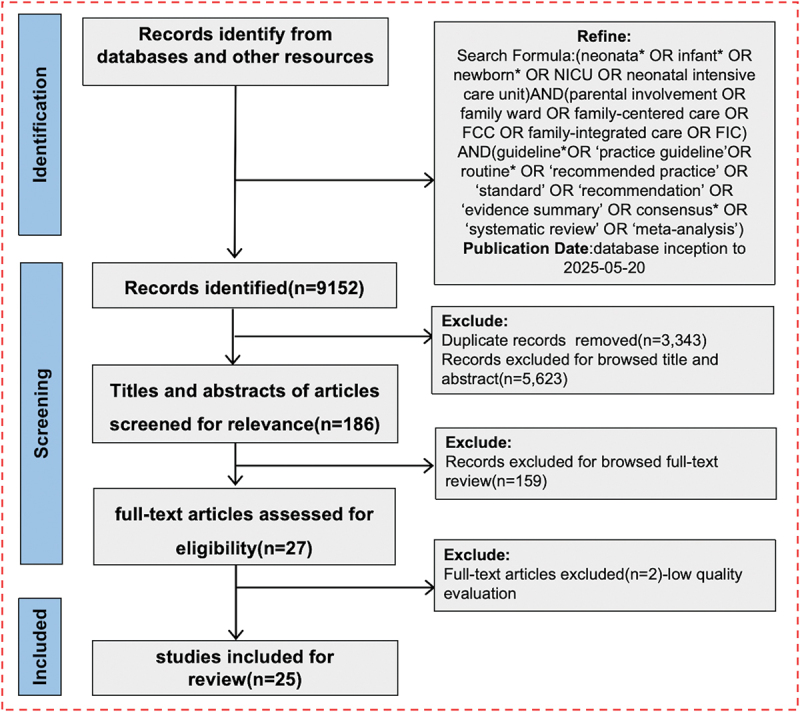
Literature search process flowchart.

The initial search identified 9,152 articles. After removing duplicates and screening titles, abstracts, and full texts for eligibility according to predefined criteria, 25 publications were ultimately included. These publications comprised 3 guidelines [20–22], 3 best practice documents [23–25], 4 experts consensus statements [26–29], and 15 systematic reviews or meta-analyses [30–44]. The study selection process is illustrated in Figure 2, and the main characteristics of the included studies, including publication year, country, and evidence type, are summarized in Table 1.

**Table 1.** General information of the included literature.

Included literature	Year	Literature sources	Type of evidence	Topic of the literature
The Group of Neonatology, Society of Pediatrics, Chinese Medical Association et al.	2023	CNKI	Guideline	Guidelines for family integrated care in neonatal intensive care unit
Davidson et al.	2007	Pubmed	Guideline	Clinical practice guidelines for support of the family in the patient-centered intensive care unit: American College of Critical Care Medicine Task Force 2004–2005
Davidson et al.	2017	Pubmed	Guideline	Guidelines for Family-Centered Care in the Neonatal, Pediatric, and Adult ICU
Govindaswamy et al.	2019	Pubmed	Best practice	Needs and stressors of parents of term and near-term infants in the NICU: A systematic review with best practice guidelines
Browne et al.	2020	Pubmed	Best practice	Executive summary: Standards, competencies, and recommended best practices for infant- and family-centered developmental care in the intensive care unit
Nadine et al.	2019	Pubmed	Best practice	Individualised developmental care for babies and parents in the NICU: Evidence-based best practice guideline recommendations
Craig et al.	2015	Pubmed	Expert consensus	Recommendations for involving the family in developmental care of the NICU baby
Hall et al.	2015	Pubmed	Expert consensus	Recommendations for peer-to-peer support for NICU parents
Hynan et al.	2015	Pubmed	Expert consensus	Recommendations for mental health professionals in the NICU
Hall et al.	2015	Pubmed	Expert consensus	Recommendations for enhancing psychosocial support of NICU parents through staff education and support
Bellizzi et al.	2024	Pubmed	Systematic review	Family-centered care for newborns: A global perspective and review
Ciupitu-Plath et al.	2021	Pubmed	Systematic review	Parent needs assessment instruments in neonatal intensive care units: Implications for parent education interventions
Dall’Oglio et al.	2018	Pubmed	Systematic review	A systematic review of instruments for assessing parent satisfaction with family centred care in neonatal intensive care units
Ding et al.	2019	Pubmed	Systematic review	Effects of family-centred care interventions on preterm infants and parents in neonatal intensive care units: A systematic review and meta-analysis of randomised controlled trials
Dol et al.	2017	Pubmed	Systematic review	eHealth interventions for parents in neonatal intensive care units: A systematic review
Kocakabak et al.	2024	Pubmed	Systematic review	Identifying outcomes and outcome measures in neonatal family-centered care trials: A systematic review
Kutahyalioglu et al.	2023	Pubmed	Systematic review	Effects of family-centered care on bonding: A systematic review
O’Callaghan et al.	2019	Pubmed	Systematic review	Evidence-based design for neonatal units: A systematic review
Segers et al.	2019	Pubmed	Systematic review	The impact of family centred care interventions in a neonatal or paediatric intensive care unit on parents’ satisfaction and length of stay: A systematic review
Barnes et al.	2024	Pubmed	Systematic review	Effectiveness and family experiences of interventions promoting partnerships between families and pediatric and neonatal intensive care units: A mixed methods systematic review
Shields et al.	2012	Pubmed	Systematic review	Family-centred care for hospitalised children aged 0–12 years
Yu et al.	2019	Pubmed	Systematic review	Family-centred care for hospitalized preterm infants: A systematic review and meta-analysis
van Veenendaal et al.	2020	Pubmed	Systematic review	Hospitalising preterm infants in single family rooms versus open bay units: A systematic review and meta-analysis of impact on parents
He et al.	2023	CNKI	Systematic review	Safety of family integrated care model in neonatal intensive care unit: A meta-analysis
Hodgson et al.	2025	Pubmed	Systematic review	Infant and Family Outcomes and Experiences Related to Family-Centered Care Interventions in the NICU: A Systematic Review

### Assessment outcomes of the included studies

#### Guideline quality evaluation results

Three clinical practice guidelines were appraised using the AGREE II instrument [20–22]. One guideline [21] achieved standardized scores of ≥60% across all six AGREE II domains and was therefore assigned an A-level recommendation. The remaining two guidelines [20,22] demonstrated moderate methodological quality and were assigned B-level recommendations. Detailed standardized domain scores and overall quality judgments are presented in Table 2, ensuring transparency of the appraisal process.

**Table 2.** Methodological quality evaluation results of the guidelines.

Included literature	Percentage of field standardization %						≥60% field number (*n*)	≥30% field number (*n*)	Recommendation level
	Scopes and objects	Participant	Rigor of the guidelines	Clarity of guidelines	Application of guidelines	Independence of the guide			
The Group of Neonatology, Society of Pediatrics, Chinese Medical Association et al.	100%	88.89%	85.42%	94.44%	52.08%	91.67%	5	6	B
Davidson, Aslakson et al.	88.89%	86.11%	66.67%	80.56%	62.50%	87.50%	6	6	A
Davidson, Powers et al.	91.67%	63.89%	71.88%	91.67%	47.92%	83.33%	5	6	B

Note: Standardization percentage of each field = (obtained score − minimum possible score)/(maximum possible score − minimum possible score) × 100%; Recommendation level: if the standardized percentage of six fields is >60%, it is highly recommended (level A); if >3 areas have a standardized percentage >30% and <60% are recommended (level B); if there are ≥3 areas with a standardized percentage <30%, it is not recommended (level C).

#### Systematic review quality evaluation results

Among the 15 systematic reviews and meta-analyses assessed using the JBI Critical Appraisal Checklist, 14 were rated as high methodological quality and one as moderate quality. All reviews met the minimum methodological threshold for inclusion in evidence synthesis. Detailed appraisal results for each item are reported in Supplementary Table S2.

#### Expert consensus quality evaluation results

Four expert consensus statements were independently evaluated by two reviewers using the JBI Expert Opinion Appraisal Tool. All four met predefined methodological standards and were judged to be of high overall quality. Detailed appraisal results are provided in Supplementary Table S3.

#### Best practice quality evaluation results

For the three best practice documents, the underpinning primary evidence was examined and critically appraised according to study design. All supporting evidence demonstrated high methodological quality, supporting their inclusion in the synthesis.

### Summary and description of evidence

Evidence extracted from the included publications was graded according to the JBI evidence and recommendation system. Following structured synthesis and integration, findings were categorized into seven key domains: key components of FCC/FICare models, implementation strategies, clinical outcomes, safety considerations, cultural adaptability, ethical implications, and digital health technologies. Ultimately, 28 high-quality evidence items were identified and translated into graded recommendations. Across domains, the synthesized evidence indicates that FCC and FICare are most effective when parental involvement is systematically integrated into routine NICU care. Three interrelated components consistently emerge: structured, competency-based participation in daily neonatal care; organizational and environmental readiness, including interdisciplinary collaboration, supportive infrastructure, and enabling policies; and sustained psychosocial support combined with consistent clinician–parent communication. Reported benefits extend beyond neonatal clinical indicators to include improvements in parental psychological well-being and caregiving confidence. Digital health approaches and culturally responsive strategies can enhance accessibility and contextual adaptability, while direct parental engagement remains central to effective care. These recommendations, together with their corresponding evidence levels and strength of recommendation, are summarized in Table 3. To further illustrate the impact of these practices on NICU processes and clinical/family outcomes, Supplementary Table S4 is provided.

**Table 3.** Summary of the best evidence for parental involvement in NICUs.

Evidence items		Evidence content	Level of evidence	Recommended level
Key components of FCC/FIC models	Parental education and involvement	Parents should acquire essential newborn care competencies, including hand hygiene, weight measurement, vital sign monitoring, diaper changing, urine output estimation, newborn dressing/undressing, kangaroo mother care (skin-to-skin contact), umbilical cord care, oral care, feeding techniques, newborn bathing, pain management, postural adjustment, sleep promotion, supportive environment, etc. [20,23,25,26,33].	1a	A
		Parents should be fully engaged in neonatal care and decision-making processes to assume their parental roles [30].	5b	A
	Psychosocial support	During the baby’s hospitalization, peer support can be provided to parents through various methods, including email or phone support, group support, or via an online community support platform for NICU families [28].	5b	A
		Validated tools should be used to screen emotional distress of parents during neonatal hospitalization, followed by screening conducted by healthcare home visitors after discharge and at developmental follow-up appointments [27].	5b	A
	Clinician−patient communication	Use structured communication tools (the ‘VALUE’ mnemonic): Active listening, empathy, and reassurance of non-abandonment [21].	1c	B
		Ensure multidisciplinary team coordination and standardize information across all healthcare personnel to provide consistent messaging about treatment goals, reducing conflicts and avoiding discrepancies [22,24].	4b	B
Implementation strategies	Environmental optimization	The NICU environment should prioritize physical infrastructure (e.g. handwashing stations) and family-friendly amenities (e.g. rest areas, nutrition access) [37,38].	2a	A
		Single-family rooms (SFRs) are recommended as they can improve outcomes for preterm neonatals, family satisfaction, privacy, parental presence and skin-to-skin care, resulting in fewer inappropriate stimulations such as high levels of noise and illumination [21,25,37,38].	2a	A
		It is recommended to implement 24-hour cycled lighting (rather than continuous bright light) based on the infant’s state and circadian time to facilitate the establishment of daily melatonin rhythm [25].	1a	A
	Interprofessional collaboration	Effective NICU care requires a multidisciplinary team including doctors, infant developmental specialists, specialized nurses, therapists, psychologists, social workers and spiritual advisors to address clinical, psychosocial, and developmental needs [21,26].	1c	A
		Incorporate parents and family members into the multidisciplinary team as fully engaged, indispensable partners in treatment and care [21,24–26].	1a	A
	Training of NICU team members	To effectively support parents, neonatal care staff require comprehensive and ongoing training including: (a) understanding of infant behavioral responses, (b) mastery of supportive care techniques, (c) developmental care principles, and (d) information regarding expected infant development and and the influence of the neonatal setting [25,26].	1a	A
	Transition to home	Transition planning from the NICU to home should start at admission, including: (a) discharge criteria education, (b) safety guidance (e.g. safe sleep), (c) hands-on care practice, (d) follow-up resources (e.g. home nursing visits, developmental care specialists, and breastfeeding support), and (e) assessment of parental social support system, mental health and home safety [23,26].	5b	A
	Policies support	It is imperative that NICU policies for parent support and staff support for family-centered developmental care be in place to offer the standard of care necessary for optimal outcomes of both baby and parent [26].	5b	A
		It is recommended that parents participate in FICare in the NICU for 3–4 hours daily. Well-resourced NICUs may consider extending this to 24-hour participation where feasible [20,25,26,33].	3c	A
		Implement inclusive visitation policies to allow extended family (e.g. baby’s siblings, grandparents) to visit with childcare support [21,24,26].	1c	A
Clinical outcomes	Neonatal outcomes	FICare improves infant outcomes, including weight gain, breastfeeding rates, shorter hospital/NICU stays, and rehospitalization, reduced duration of oxygen therapy, and enhanced brain structural development and psychomotor behavioral development [20,26,37,39,42,44].	1a	A
	Family outcomes	FICare/FCC interventions reduce parental stress/anxiety, strengthen parent−infant bonding, improve infant care confidence and enhance parental satisfaction [20,26,31,36,39,41,44].	1a	A
Safety considerations	Patient safety	During FICare implementation, providing real-time guidance to parents on identifying the locations of various tube labels can significantly reduce the incidence of adverse events such as ventilator disconnection and accidental tube removal [20].	3b	B
		Parents are required to abstain from alcohol and avoid fall-risk medications before and after FICare implementation to prevent sudden infant death syndrome (SIDS) [20].	3d	B
	Infection risk management	Critical measures include rigorous parental hand hygiene, extended to equipment in contact with infants (e.g. lead wires), and promotion of the ‘baby space concept’ to standardize hygiene practices [20].	1d	A
		Infrastructure design should prioritize infection prevention through optimized sink placement, airborne isolation facilities, water safety, and sustainable materials with acoustic/illuminative suitability [37].	3c	A
Digital Health Technologies		Apply eHealth (e.g. videoconferencing, internet-based applications, SMS system, etc) to improve parent and neonatal outcomes (particularly around parent acceptance and use), and enhance clinician-family education and communication [34].	1c	A
		Telemedicine can be used for both screening and treatment, potentially increasing accessibility for families in low resource settings [27].	1c	A
Cultural Adaptation		Healthcare systems should integrate cultural competence training for providers and align provider-patient cultural backgrounds to mitigate conflicts [22].	4c	A
		When communicating with non-English-speaking families, utilizing trained non-volunteer interpreters facilitates the establishment of effective clinician-patient communication and enables healthcare teams to better comprehend the influence of cultural norms [29].	3c	A
Ethical Implications		Integrate dedicated pastoral care personnel within NICU teams to enhance staff self-awareness of values, particularly how their religious background and spiritual perspective may affect their interactions with patients [29].	4d	B
		NICU staff should receive both initial and ongoing training and education in ethics, including palliative care consultation and ethics consultation for critically ill patients with clinician-family value conflicts to reduce ICU and hospital LOS [21,29].	1b	A

Note: *Consider, if feasible to do so, reporting the number of records identified from each database or register searched (rather than the total number across all databases/registers).

**If automation tools were used, indicate how many records were excluded by a human and how many were excluded by automation tools.

Source: Page MJ, et al. BMJ 2021;372:n71. doi: https://10.1136/BMJ.n71.

This work is licensed under CC BY 4.0. To view a copy of this license, visit https://creativecommons.org/licenses/by/4.0/.

## Discussion

### Significance and validity of evidence on parental involvement

Parental involvement in NICUs is a dynamic and modifiable factor that substantially influences both infant and family outcomes [20]. Structured engagement strategies, including competency-based education, psychosocial support, and active participation in daily neonatal care, enhance infant growth, feeding, and neurodevelopment while simultaneously improving parental confidence and reducing stress [36,37]. Beyond these individual benefits, early and consistent parental involvement strengthens collaboration between families and healthcare teams, facilitates shared decision-making, and promotes relational continuity. In this review, a systematic search identified 25 relevant publications, from which 28 best evidence points were extracted across seven domains. Analysis of these findings indicates that parental involvement is most effective when integrated into routine NICU workflows rather than treated as an optional component. Coordinated education, continuous psychosocial support, and clear clinician–parent communication emerge as critical factors that determine the success and sustainability of family-centered and family-integrated care. Collectively, these findings underscore that embedding parental engagement as a standard practice is essential for achieving both optimal neonatal outcomes and improved family well-being.

### Core components of FCC and FICare models in NICUs

FCC and FICare share core values of family engagement, collaborative care, and respect for parental role [30]. The effectiveness of these models arises from systematically integrating parents into daily care rather than relying solely on their physical presence. While FICare defines more structured parental roles, both models converge on three interrelated domains: education, psychosocial support, and clinician–parent communication. Structured education programs in NICUs are consistently associated with enhanced parental competence, confidence, and preparedness for discharge. A Canadian pilot initiative for mothers of preterm infants combined daily teaching sessions, bedside caregiving, and participation in medical rounds. This approach demonstrated that skill acquisition and direct engagement reinforce caregiving self-efficacy and readiness for home care [45]. Similarly, the HUG Your Baby program in a Level II Special Care Nursery (SCN) showed that targeted education reduced maternal stress and improved confidence, indicating that structured learning mediates psychosocial benefits and facilitates sustained parental involvement [46].

Psychosocial support directly influences both parental well-being and infant outcomes. Families frequently experience prolonged stress during NICU hospitalization [27]. Evidence indicates that structured peer support and integrated mental health services reduce parental isolation, enhance coping, and strengthen parent–infant interaction, with potential downstream effects on healthcare utilization and costs [47,48]. Screening for depressive symptoms among mothers and fathers is necessary, yet effectiveness depends on timely assessment and integration into routine care. Collaborative care models embedded in NICU workflows mitigate referral barriers and attenuate long-term psychosocial risks, demonstrating that psychosocial interventions are most impactful when operationalized systematically [48]. Consistent and empathetic communication amplifies the benefits of parental involvement. Frameworks such as the VALUE mnemonic provide structured guidance, including active listening, emotional reassurance, and explicit acknowledgment of parental role [21]. Evidence indicates that clear and sensitive communication enhances shared decision-making, strengthens trust, and supports parental self-management. Communication functions as a critical mechanism linking educational and psychosocial interventions to measurable outcomes for both infants and their families [49]. Together, these components reveal that FCC and FICare operate as system-level interventions in which structured education, psychosocial support, and effective communication interact synergistically. This integrated approach shifts care from passive family presence to active engagement, producing cascading benefits for infant clinical outcomes, parental psychological well-being, and overall NICU relational quality.

### Practical strategies for implementing family-inclusive care

Effective implementation of family-inclusive care requires a coordinated, multifaceted approach addressing environmental, organizational, educational, and policy dimensions. The physical environment is a key determinant of parental engagement and infant outcomes. Excessive noise, bright lighting, and temperature fluctuations elevate infant stress and reduce parent–infant interaction, whereas thoughtfully designed spaces that provide comfort, privacy, and areas for rest and skin-to-skin contact enhance developmental support and promote sustained involvement [50]. Single-family rooms (SFRs) illustrate how infrastructure influences outcomes. SFRs provide privacy, reduce disruptions, lower infection rates, and may decrease reliance on parenteral nutrition and invasive procedures, supporting both infant recovery and parental confidence [51]. Interdisciplinary teamwork is essential to address the complex medical, developmental, and psychosocial needs of infants and their families [21,26]. Evidence from a Thai NICU demonstrates that staff endorsement of FCC principles does not always translate into practice, highlighting the role of organizational culture and attitudes in mediating effectiveness [52].

Staff education is critical for operationalizing family-inclusive care. Training programs that enhance knowledge of infant cues, developmentally supportive care, and long-term NICU implications produce measurable improvements in staff competence [53]. Complementary reflective practice and ongoing professional development in ethics, self-awareness, and cultural sensitivity enable staff to navigate complex interactions and support equitable engagement. These staff-focused strategies ultimately aim to enhance parental education and participation in neonatal care while ensuring that parents are effectively empowered to engage in their infant’s care [54]. Early discharge planning integrates hospital-based interventions with home preparation, including education on discharge criteria, hands-on caregiving skills, available support services, and assessment of family readiness and psychological well-being [23,26]. A randomized trial in Turkey showed that structured FICare education delivered by trained nurses improved parental confidence in caregiving, feeding, and breastfeeding while promoting infant weight gain, particularly among high-risk infants [55]. These findings demonstrate how embedding structured educational interventions into daily workflows translates into improved parental competence and infant outcomes. Supportive institutional policies are foundational. Unrestricted parental access eliminates perceived barriers, reinforces engagement, and aligns with international guidelines emphasizing maternal–infant co-location [50]. Collectively, these strategies demonstrate that effective implementation emerges from the interaction of the environment, staff expertise, educational programming, and policies. Each element reinforces the others, producing synergistic benefits extending from infant outcomes to parental confidence, family functioning, and overall well-being.

### Impact on infant and parental clinical outcomes

FICare and FCC interventions provide consistent and measurable benefits for infants and families. Randomized studies show that structured parental involvement accelerates attainment of full enteral feeding, promotes weight gain, increases exclusive breastfeeding, shortens hospital stays, and reduces complications and readmissions [10]. These outcomes suggest that parental participation enhances physiological stability and care efficiency through mechanisms, such as better feeding regulation, improved thermoregulation during skin-to-skin contact, and responsive caregiving. Although cognitive or language outcomes may not uniformly improve by 18 months, evidence indicates enhanced motor development and physical growth, as measured by higher Bayley-III motor scores and body mass index [56]. This pattern suggests that early engagement supports fundamental growth trajectories, while some neurodevelopmental domains may require longer-term follow-up.

From a family perspective, FICare reduces parental stress and anxiety while increasing confidence and caregiving competence. These benefits are driven by skill acquisition, structured support, and integration into daily care routines, fostering parental self-efficacy and strengthening parent–infant bonding [57,58]. Fathers also experience lower stress and greater emotional connection through active participation [57]. Structured FICare education enhances maternal problem-solving and coping within the NICU context [58]. These findings highlight a dual pathway: parental engagement improves infant clinical outcomes and simultaneously enhances parental psychological well-being and family functioning [10]. This perspective emphasizes that benefits arise mechanistically from structured roles, interdisciplinary support, and routine integration rather than incidental effects. Understanding these mechanisms is essential for designing scalable interventions that optimize outcomes across diverse neonatal populations.

### Safety, cultural, and ethical considerations in practice

Effective FICare and FCC implementation depends on organizational, cultural, and ethical factors. Organizational culture strongly influences parental involvement, with inclusive and collaborative units demonstrating higher adoption and fidelity, whereas rigid hierarchies and inconsistent policies create barriers [59]. Successful implementation requires alignment of institutional norms, staff training, and communication practices to support parental participation as a core component of care. Ethical challenges arise when disparities in staff education, information sharing, or institutional messaging limit equitable engagement or informed consent. During the COVID-19 pandemic, visitation restrictions in southern Thailand increased parental stress and disrupted communication, illustrating how external constraints interact with institutional policies to affect both ethics and feasibility [60]. Policies must be adaptable to preserve safety while maintaining meaningful parental engagement.

Cultural factors also shape care effectiveness. Evidence from California NICUs indicates that families from marginalized backgrounds experience systemic barriers, including limited shared decision-making, inconsistent nurse–parent continuity, and restricted access to interpretation services [61]. Culturally responsive strategies, such as communication training and interpreter integration, are essential for equitable implementation. Safety, ethics, and cultural responsiveness are interdependent determinants of FCC/FICare success. Institutional support, policy flexibility, and culturally competent practices collectively enhance fidelity and quality of engagement, mitigating risks and optimizing outcomes across diverse contexts.

### Contextual adaptation and evidence gaps in sub-Saharan Africa

Although our review did not identify high-level synthesized evidence on FCC or FICare implementation in sub-Saharan Africa, emerging regional research offers important insights into current practices and contextual constraints. Evidence from South Africa, for example, highlights the value of culturally responsive neonatal care. A qualitative study conducted in Limpopo Province described parents’ experience of caring preterm infants in NICUs and underscored the need to integrate indigenous beliefs and practices strengthening parental engagement [62]. Findings from Uganda suggest that parental involvement frequently takes the form of task-oriented caregiving rather than true partnership. Healthcare providers attributed this pattern to resource limitations and heavy workloads, which shape how parents are incorporated into neonatal care processes [63]. Similarly, research in Ghana has identified multiple structural and interpersonal barriers to effective FCC implementation, including parental stress, limited information sharing, restrictive access policies, and staffing shortages [64]. Data from Rwanda further illustrate both existing engagement and unrealized potential. In two public neonatal units, parents were already performing essential caregiving activities, such as feeding, hygiene, and bonding. Many expressed willingness to assume broader responsibilities if supported through structured education and clear guidance [65]. Together, these studies indicate that although the formal FCC and FICare frameworks remain under-evaluated in sub-Saharan Africa, the foundational principles of parental participation and culturally attuned care are gaining recognition. Advancing context-specific adaptation and generating high-quality evidence will be critical to ensuring equitable and feasible implementation of family-centered and integrated care models in resource-constrained settings.

### Opportunities and challenges in integrating digital health tools

Digital health tools have been extensively used as adjuncts in FICare, particularly under constraints, such as the COVID-19 pandemic. These technologies can enhance parental engagement by providing access to information, supporting remote participation, and facilitating continuous parent–infant interaction [66]. However, effectiveness depends on usability, credibility, and integration into care workflows. Only a minority of NICU-targeted applications meet quality standards, raising concerns about inconsistent content, misinformation, and variable impact on parental confidence [67]. Video communication platforms offer both opportunities and challenges. Live video calls provide real-time reassurance but may increase parental anxiety and staff workload [49]. Asynchronous messaging mitigates these challenges by allowing flexible engagement, supporting emotional well-being, fostering trust, and reinforcing participation [68]. Bibliometric evidence indicates that digital integration in neonatal care is expanding, with focus areas including parental engagement, artificial intelligence, and telehealth [69]. Analysis shows that digital tools cannot replace core FICare components, such as bedside presence and skin-to-skin care; their role is complementary, amplifying relational and hands-on care. Effective integration requires alignment with institutional policies, staff training, and culturally responsive practices to ensure equitable access and sustained benefit.

### Limitations and future directions

This review provides a comprehensive synthesis of the best available evidence on parental involvement in NICUs; however, several limitations must be acknowledged. First, the search timeframe (up to May 2025) may have excluded recently published or emerging studies. Second, focusing exclusively on guidelines, systematic reviews, and expert consensus may introduce selection bias, as it overlooks primary intervention studies and gray literature that capture real-world implementation challenges. Third, the included evidence predominantly originates from high-income settings, and limiting publications to English and Chinese may restrict the generalizability of findings to low-resource contexts, where family engagement models, infrastructure, and available support differ substantially. Future research should address these gaps by evaluating long-term neurodevelopmental outcomes of FCC and FICare through longitudinal studies. Context-specific cost-effectiveness analyses across diverse resource settings are needed, alongside the development of culturally responsive, co-designed care models tailored to local caregiving practices. Scalable implementation strategies will benefit from hybrid effectiveness – implementation trials, continuous monitoring of infant, parent, and staff outcomes, and standardized metrics of family engagement and equity. Adopting a multifaceted approach will facilitate the creation of precision FCC/FICare models that integrate biological, psychosocial, and sociocultural considerations across NICUs globally.

## Conclusions

This evidence summary demonstrates that FCC and FICare are conceptually aligned and deliver measurable benefits to infants and families, including improved clinical outcomes, enhanced parental confidence, and reduced psychological stress. Effectiveness depends on structured parental education, consistent psychosocial support, optimized NICU environments, and coordinated interdisciplinary care, rather than isolated interventions. Digital tools may facilitate engagement but are most effective when integrated with relational, presence-based care. The predominance of evidence from high-resource settings highlights the need for contextual adaptation to ensure equity and feasibility in diverse NICUs. Overall, these findings emphasize the importance of moving beyond descriptive reporting. They highlight the need for precision and context-sensitive implementation strategies that reliably translate FCC/FICare principles into practice worldwide.

## Supplementary Material

GHA_Supplementary_Tables_de.docx

PRISMA_2020_Flow_Diagram.docx

PRISMA_2020_Checklist.docx

## Data Availability

The data for this study were derived from publicly available sources, including clinical guidelines, evidence summaries, expert consensus statements, systematic reviews, and meta-analyses identified through a structured literature search following the 6S evidence model. All data analysed during this study are included in this published article. Additional information supporting the findings of this review is available from the corresponding author upon reasonable request.
