# Supplementary material for: Summary of best evidence on parental involvement in neonatal intensive care units: integrating family-centered and family-integrated care models
Source: Glob Health Action. 2026 May 5;19(1):2653286. doi: 10.1080/16549716.2026.2653286 (PMC13148075; doi:10.1080/16549716.2026.2653286)
Supplement: GHA_Supplementary_Tables_de.docx [file ZGHA_A_2653286_SM8458.docx]

**Supplementary Table S1**. Detailed Methodological Workflow for Evidence Summary

| **Step** | **Action** | **Tool / Framework** | **Personnel / Decision Process** | **Notes / Rationale** |
| --- | --- | --- | --- | --- |
| Evidence Identification | Comprehensive search of guideline repositories, evidence-based platforms, and major literature databases | BMJ Best Practice, UpToDate, NICE, AHRQ, GIN, CMA Infobase, SIGN, CGC, WHO, NZGG, RNAO, Trip Database, JBI, Cochrane Library, PubMed, CINAHL, Web of Science, CNKI, Wanfang, VIP, Sinomed | Conducted by trained members of the review team | MeSH and free-text terms combined for NICU, parental involvement, FCC/FICare, and evidence type; timeframe: inception to 20 May 2025; ensures comprehensiveness and reproducibility |
| Eligibility Screening | Apply predefined inclusion and exclusion criteria | Manuscript-defined criteria | Two independent reviewers; disagreements resolved by third reviewer | Inclusion: neonates admitted to NICUs; parental involvement, FCC, or FICare; high-level evidence, publications in English or Chinese.Exclusion: duplicate records; articles of the wrong document type; inaccessible full texts; topics not relevant to the study focus. |
| Quality Appraisal | Critical assessment of methodological rigor | AGREE II (guidelines), JBI Checklist (systematic reviews/meta-analyses), JBI Expert Consensus Standards | Two independent reviewers; third reviewer adjudicated discrepancies | High-quality studies prioritized; ensures reliability and transparency in synthesis |
| Data Extraction | Standardized collection of study characteristics and outcomes | Predefined extraction form | Two independent reviewers; blinded to each other | Extracted: author, year, country, evidence type, study focus, key findings, recommendations |
| Evidence Synthesis | Organize findings into thematic domains | Thematic synthesis | Entire review team | Domains: core FCC/FICare components, implementation strategies, clinical outcomes, safety, cultural adaptability, ethical considerations, digital health tools; iterative refinement ensures coherence and clinical relevance |
| Conflicting Evidence | Resolve discrepancies across sources | Quality prioritization | Two independent reviewers; third reviewer resolved disagreements | Evidence with higher methodological quality, stronger evidence levels, and more recent publication dates prioritized; rationale documented |
| Recommendation Development | Consolidate actionable statements from synthesized evidence | JBI FAME framework (Feasibility, Appropriateness, Meaningfulness, Effectiveness) | Structured team discussions | Each recommendation linked to supporting evidence, graded A (strong) or B (weak); overlapping/redundant recommendations merged to enhance clarity |
| Transparency & Traceability | Document all methodological steps | Supplementary table | Entire review team | Provides reproducibility and transparency for the scientific community; traceability from evidence identification → appraisal → synthesis → final recommendations |

**Supplementary Table 2** Methodological quality evaluation results of systematic review

| Systematic review | Evaluation entry | | | | | | | | | | | Overall quality |
| --- | --- | --- | --- | --- | --- | --- | --- | --- | --- | --- | --- | --- |
|  | ① | ② | ③ | ④ | ⑤ | ⑥ | ⑦ | ⑧ | ⑨ | ⑩ | ⑪ |  |
| Bellizzi et al. | Yes | Yes | Yes | Yes | Yes | Yes | Yes | Yes | No | Yes | No | High |
| Ciupitu-Plath et al. | Yes | Yes | Yes | Yes | Yes | Yes | Yes | Yes | No | Yes | Yes | High |
| Dall’Oglio et al. | Yes | Yes | Yes | Yes | Unclear | Yes | Yes | Yes | No | Yes | Yes | High |
| Ding et al. | Yes | Yes | Yes | Yes | Yes | Yes | Yes | Yes | No | Yes | Yes | High |
| Dol et al. | Yes | Yes | Yes | Yes | Yes | Yes | Yes | Yes | No | Yes | Yes | High |
| Kocakabak et al. | Yes | Yes | Yes | Yes | Yes | Yes | Yes | Yes | No | No | Yes | High |
| Kutahyalioglu et al. | Yes | Yes | Yes | Yes | Yes | Yes | Yes | Yes | No | Yes | Yes | High |
| O’Callaghan et al. | Yes | Yes | Yes | Yes | Yes | Yes | Unclear | Yes | No | Yes | No | Medium |
| Segers et al. | Yes | Yes | Yes | Yes | Yes | Yes | Yes | Yes | No | Yes | Yes | High |
| Barnes et al. | Yes | Yes | Yes | Yes | Yes | Yes | Yes | Yes | No | Yes | Yes | High |
| Shields et al. | Yes | Yes | Yes | Yes | Yes | Yes | Yes | Yes | No | Yes | Yes | High |
| Yu et al. | Yes | Yes | Yes | Yes | Yes | Yes | Yes | Yes | No | Yes | Yes | High |
| van Veenendaal et al. | Yes | Yes | Yes | Yes | Yes | Yes | Yes | Yes | No | No | Yes | High |
| He et al. | Yes | Yes | Yes | Yes | Yes | Yes | Yes | Yes | Yes | Yes | No | High |
| Hodgson et al. | Yes | Yes | Yes | Yes | Yes | Yes | Yes | Yes | No | Yes | Yes | High |

Note: ① Is the review question clearly and explicitly stated?② Were the inclusion criteria appropriate for the review question? ③ Was the search strategy appropriate? ④ Were the sources and resources used to search for studies adequate? ⑤ Were the criteria for appraising studies appropriate? ⑥Was critical appraisal conducted by two or more reviewers independently? ⑦ Were there methods to minimize errors in data extraction? ⑧ Were the methods used to combine studies appropriate?⑨ Was the likelihood of publication bias assessed? ⑩ Were recommendations for policy and/or practice supported by the reported data?⑪Were the specific directives for new research appropriate?

**Supplementary Table 3** Methodological quality evaluation of expert consensus

| Expert consensus | Evaluation entry | | | | | | Overall quality |
| --- | --- | --- | --- | --- | --- | --- | --- |
|  |  |  |  |  |  |  |  |
| Craig et al. | Yes | Yes | Yes | Yes | Yes | No | High |
| Hall,Ryan et al. | Yes | Yes | Yes | Yes | Yes | No | High |
| Hynan et al. | Yes | Yes | Yes | Yes | Yes | Yes | High |
| Hall,Cross et al. | Yes | Yes | Yes | Yes | Yes | No | High |

Note:① Is the source of the opinion clearly stated?② Are the opinions from influential experts in the field? ③Are the opinions presented centered on the interests of the people involved in the study? ④Is the stated conclusion based on the results of the analysis? Are opinions expressed logically? ⑤ Whether to refer to other existing literature? ⑥ Are there any inconsistencies between the opinions presented and the previous literature?

**Supplementary Table S4**. Impact of Best Evidence on NICU Processes and Outcomes

| **Evidence Item** | **Impact on NICU Processes** | **Impact on Clinical / Family Outcomes** |
| --- | --- | --- |
| **Parental Education and Involvement** | Parents acquire essential newborn care skills, actively participate in routine care, and engage in decision-making; enhances staff-parent communication and workflow efficiency | Improved infant growth, feeding, neurodevelopment; increased parental confidence, reduced stress |
| **Psychosocial Support** | Integration of peer support programs, mental health screening, and structured interventions into NICU workflow | Reduced parental isolation, improved coping, enhanced parent-infant interaction, potential reduction in healthcare utilization and costs |
| **Clinician-Patient Communication** | Structured communication tools (e.g., VALUE mnemonic) standardize dialogue, facilitate multidisciplinary coordination, and reduce conflicts | Strengthened trust, enhanced shared decision-making, improved parental self-management and engagement |
| **Environmental Optimization** | NICU design prioritizes privacy, comfort, hygiene, and developmental support (e.g., SFRs, cycled lighting) | Reduced noise and stress, enhanced parent-infant interaction, improved infant physiological stability and recovery |
| **Interprofessional Collaboration** | Multidisciplinary teams incorporate parents as full participants; improved care coordination | Holistic management of medical, psychosocial, and developmental needs; improved infant outcomes and parental satisfaction |
| **Training of NICU Team Members** | Staff develop competency in infant cues, developmentally supportive care, and family engagement | Enhanced staff ability to support parents, resulting in increased parental confidence and effective care delivery |
| **Transition to Home** | Early discharge planning integrated into NICU workflow; parent education and hands-on practice standardized | Smoother home transition, improved parental competence, enhanced infant safety and care continuity |
| **Policies Support** | Standardized family-centered policies and inclusive visitation protocols; formalized parent participation schedules | Greater parental presence, sustained engagement, improved parent satisfaction, reduced stress/anxiety |
| **Neonatal Outcomes** | Implementation of structured parental involvement as routine practice | Increased weight gain, breastfeeding rates, shorter hospital/NICU stays, reduced rehospitalization and oxygen therapy duration, improved brain and psychomotor development |
| **Family Outcomes** | Embedding psychosocial support and parental engagement into daily workflows | Reduced parental stress and anxiety, stronger parent-infant bonding, improved confidence and satisfaction |
| **Patient Safety** | Real-time guidance and safety protocols for parents; standardized procedures | Reduced incidence of adverse events (ventilator disconnection, tube removal, SIDS prevention) |
| **Infection Risk Management** | Rigorous hygiene protocols, optimized infrastructure and equipment placement | Reduced infection risk and hospital-acquired complications |
| **Digital Health Technologies** | Integration of telemedicine and eHealth platforms for parent education and communication | Enhanced parental engagement, accessibility, and adherence; improved parent-infant interaction when physical presence is limited |
| **Cultural Adaptation** | Cultural competence training for staff; interpreter integration | Reduced communication barriers, equitable care delivery, increased family satisfaction |
| **Ethical Implications** | Ethics education and pastoral support integrated into NICU workflow | Enhanced staff self-awareness, improved ethically consistent decision-making, reduced ICU/hospital length of stay, better family engagement |
