# Supplementary material for: Summary of best evidence on parental involvement in neonatal intensive care units: integrating family-centered and family-integrated care models
Source: Glob Health Action. 2026 May 5;19(1):2653286. doi: 10.1080/16549716.2026.2653286 (PMC13148075; doi:10.1080/16549716.2026.2653286)
Supplement: PRISMA_2020_Flow_Diagram.docx [file ZGHA_A_2653286_SM8457.docx]

**Identification of studies via other methods**

**Identification of studies via databases and registers**

Records identified from:

Websites (n =890 )

Organisations (n =0 )

Citation searching (n =0 )

Records removed *before screening*:

Duplicate records removed (n =3343 )

Records marked as ineligible by automation tools (n =0 )

Records removed for other reasons (n = 0)

Records identified from*:

Databases (n = 8262)

Registers (n = 0)

**Identification**

Records screened

(n =4919 )

Records excluded based on title and abstract(n =4826 )

Reports not retrieved

(n = 0)

Reports sought for retrieval

(n =890 )

Reports sought for retrieval

(n =93 )

Reports not retrieved

(n =0 )

**Screening**

Reports assessed for eligibility

(n =0 )

Reports excluded:

Not relevant (n =797 )

Wrong document type(n =93)

Reports assessed for eligibility

(n =93 )

Reports excluded:

Low quality (n =2 )

Wrong document type(n =66 )

Studies included in review

(n =25 )

Reports of included studies

(n =25 )

**Included**

*Consider, if feasible to do so, reporting the number of records identified from each database or register searched (rather than the total number across all databases/registers).

**If automation tools were used, indicate how many records were excluded by a human and how many were excluded by automation tools.

Source: Page MJ, et al. BMJ 2021;372:n71. doi: 10.1136/bmj.n71.

This work is licensed under CC BY 4.0. To view a copy of this license, visit <https://creativecommons.org/licenses/by/4.0/>
